# Importance of Estrogenic Signaling and Its Mediated Receptors in Prostate Cancer

**DOI:** 10.3390/ijms17091434

**Published:** 2016-08-31

**Authors:** Kin-Mang Lau, Ka-Fai To

**Affiliations:** Department of Anatomical and Cellular Pathology, State Key Laboratory of Oncology in Southern China, and Sir YK Pao Centre for Cancer, The Chinese University of Hong Kong, Hong Kong, China

**Keywords:** prostate cancer, estrogens, antiestrogens, estrogen receptors, ERα, ERβ, GPR30, GPER, fulvestrant

## Abstract

Prostate cancer (PCa) treatment was first established by Huggins and Hodges in 1941, primarily described as androgen deprivation via interference of testicular androgen production. The disease remains incurable with relapse of hormone-refractory cancer after treatments. Epidemiological and clinical studies disclosed the importance of estrogens in PCa. Discovery of estrogen receptor ERβ prompted direct estrogenic actions, in conjunction with ERα, on PCa cells. Mechanistically, ERs upon ligand binding transactivate target genes at consensus genomic sites via interactions with various transcriptional co-regulators to mold estrogenic signaling. With animal models, Noble revealed estrogen dependencies of PCa, providing insight into potential uses of antiestrogens in the treatment. Subsequently, various clinical trials were conducted and molecular and functional consequences of antiestrogen treatment in PCa were delineated. Besides, estrogens can also trigger rapid non-genomic signaling responses initiated at the plasma membrane, at least partially via an orphan G-protein-coupled receptor GPR30. Activation of GPR30 significantly inhibited in vitro and in vivo PCa cell growth and the underlying mechanism was elucidated. Currently, molecular networks of estrogenic and antiestrogenic signaling via ERα, ERβ and GPR30 in PCa have not been fully deciphered. This crucial information could be beneficial to further developments of effective estrogen- and antiestrogen-based therapy for PCa patients.

## 1. Prostate Cancer Epidemiology

Prostate cancer (PCa) is the most common solid malignancy and the second leading cause of cancer-related death in men in the United States, posing significant impacts on men’s health. The American Cancer Society estimates for 2016 that approximately 180,890 new cases will be diagnosed and 26,120 men will die of the disease, accounting for about 8.3% of male cancer-related deaths (American Cancer Society, Cancer Statistics Center [1]). This cancer is extremely rare before the age of 40 but the incidence increases with age. The rate of increase with age is higher than those for any other cancers and it increases at approximately the 9th–10th power of age [2]. In addition, race is also an important risk factor. African-American men have the highest rates in the world at about 50%–70% higher than in Caucasian-Americans [3,4,5,6,7,8,9,10,11]. For Asian populations, native Chinese and Japanese have the lowest PCa rates [4,8,12,13]. The differences in the incidences was supposed to be related to different detection strategies for PCa. However, after adjusting these diagnostic biases, there are still significant differences among populations [8,12,14]. Even though the incidence rates of Chinese- and Japanese-Americans increase after they emigrated to America as compared to those in their homeland, the rates are still much lower than that of Caucasians and African-Americans [14,15,16,17], indicating the importance of intrinsic contributing factors to this disease.

Regardless of the incidence rates of symptomatic PCa, there are no significant differences for microscopic cancers (i.e., latent cancers) among races [18,19,20,21]. These asymptomatic cancers were frequently found in elderly men either at autopsy or after cryoprostatectomy for various pathological conditions of their bladders. It is usually small, with a <0.5 mm diameter, and well differentiated [20,22,23,24]. Similar prevalence of this latent cancer among racial groups suggests that PCa initiation is likely due to endogenous factors, although the promotion step leading to clinical symptomatic cancer is predominantly influenced by exogenous factors [25]. It was speculated that endogenous estrogens such as estrone and estradiol and exogenous estrogens, e.g., phytoestrogens and xenoestrogens, dictate the estrogenic signaling in the prostate to modulate the promotion step for clinical cancer developments [25].

## 2. Epidemiological and Clinical Evidence of Hormonal Involvements in Prostatic Carcinogenesis

Epidemiological studies of hormone profiles of populations with different degrees of PCa risk demonstrated that high serum levels of estrogens were associated with high risk and thus this steroid hormone might be one of the risk factors [3,26,27,28,29]. It was reported that the levels of estrone and estradiol in African old men are higher than those in Caucasian [26,30,31,32]. In addition, serum levels of estradiol were 15% higher in Caucasian-Dutch men than in Japanese men [28]. Regarding prenatal exposures, pregnant African-American women have 37% higher circulating estradiol levels than European-American women, indicating higher exposure to estrogens in African-American men than European-American before birth [27,29]. However, population-based case-control studies [33,34,35,36,37] generally showed no significant differences in serum levels of estrogens between cancer patients and control subjects, except Barrett-Connor et al.’s study that the incidence rate linearly increased with increasing serum level of estradiol [38].

Furthermore, the incidence of PCa sharply increases in aging men at a time when the ratio of estrogens to androgens showed a 40% increase. As men age, their plasma levels of testosterone and other androgenic hormones decrease due to declining testicular functions [25,39,40,41,42,43]. Concurrently, the prostatic epithelial cells but not stromal cells have lower levels of 5α-hydrotestosterone (DHT) [44] although the level in the whole prostate showed no change [45] in accordance with the previous findings of the decreased DHT-forming index (i.e., *V*_max_/*K*_m_ of 5α-reductase) with age [46]. Concurrently, serum estrogens in aging men increase because of active aromatization of adrenal androgens to estrogens in their peripheral adipose tissues [25,39,40,41,42,47]. These endocrine changes at men’s mid-life are referred as an andropause. Besides, high cellular levels of estrone and estradiol are observed in the aged prostate [44]. Taken all together, the changes of hormonal stimulations in the aged prostate, especially increased estrogenic stimulation, may contribute to prostatic carcinogenesis.

## 3. Estrogen- and Androgen-Induced Prostatic Lesions in Animals

Administration of pharmacological doses of estrogens had been reported to induce squamous metaplasia (i.e., a proliferative activity of basal cells with transdifferentiation into any intermediate stage of differentiation between squamous cells, luminal secretory cells and basal cells in epithelium) in regressed prostates of castrated or hypophysectomized dogs [48,49]. Levine and colleagues also observed this estrogen-induced squamous metaplasia in men who had benign prostatic hyperplasia and underwent medical castration therapy with gonadotropin-releasing hormone agonist for more than six months [50]. The metaplastic change is initiated by proliferation of basal cells which subsequently differentiate into squamous cells [48,49,50]. On the contrary, administration of estrogen in castrated guinea pigs induced hypertrophy of secretory cells in the lateral prostate and increased the thickness of fibromuscular layer, possibly due to the increased cell proliferation of smooth muscle cells [51,52,53,54]. Different from dogs, only the seminal vesicle showed basal cell hyperplasia in the estrogen-treated castrated guinea pigs [52,53]. For the intact mice, a synthetic estrogen diethylstilbestrol provoked epithelial squamous metaplasia in the prostate [55]. These indicated that hormonal backgrounds, likely androgenic stimulations in the intact animals, are crucial in directing the cell fate of estrogenic responses in the prostate.

When Wistar rats were prenatally and/or neonatally exposed to estrogens, they developed squamous cell carcinoma in the prostate [56,57,58,59]. Prenatal [59] and neonatal [60] estrogenic exposures induced hyperplasia and dysplasia in the prostates respectively. It was reported that increase of free-serum estradiol in male murine fetuses though a maternal Silastic estradiol implant induced prostate enlargement in the adult mice with increased androgen sensitivity [61]. The sensitivity to androgens in the adult prostate was also enhanced by a low-dose treatment of estradiol in the immature male rat at 20–22 days of age. This permanent alteration of the prostate by neonatal estrogen treatment was described as “estrogen imprinting” [62]. Comprehensive studies of estrogen imprinting in rat prostates demonstrated a decrease in weight, alterations of DNA contents and morphological changes in three prostatic lobes (ventral, lateral and dorsal) of neonatal estrogenized rats [63,64]. The hypoplastic ventral and dorsal prostates showed an increase of interacinar stromal tissues, epithelial hyperplasia in disorganized acini, luminal sloughing and apparent lack of differentiation as well as a decrease in the levels of androgen and estrogen receptors [63,64,65].

Treatment of estradiol in intact Noble rats caused massive atrophy in the prostate [66,67]. This may be due to suppression of LHRH stimulation of the pituitary gland by the estrogens. The treatment indirectly decreases production of testicular testosterone. As the prostate largely depends on androgens for its normal growth and functions, reduction of androgen level results in the regression [66,67]. Combined treatment of testosterone and estradiol induced PCa in Noble rats after 32 weeks with 100% induction rate [66,68,69,70,71,72]. The cancers were derived from the periurethral, proximal ducts of the dorsolateral and anterior prostates of the rats [66,67,72]. The induction can also be achieved in a shorter period of time by an extremely high dose of testosterone in combination with estradiol [73]. In fact, long-term androgen, either testosterone or 5 α-dihydrotestosterone, treatment alone was able to induce PCa but the incidence was low [66,67,68,69,74]. Taken together, testosterone is likely a weak carcinogen but estradiol in combination can potentiate these carcinogenic effects in the prostate, implying the significance of estrogens in prostatic carcinogenesis. Interestingly, the estradiol plus testosterone treatment-induced PCa in Noble rats was confined to the periurethral, proximal ducts of the dorsolateral and anterior prostates but not in the periphery of the glands where dysplasia developed in the acini [66,67,72]. Histologically, the acinar dysplasia in the periphery of the rat prostate is similar to human prostatic intraepithelial neoplasia (PIN) which is considered to be a precursor of prostatic adenocarcinoma [66,67,72,75,76,77,78,79,80,81]. In addition to the Noble rats, the combined treatment induced atypical hyperplasia and carcinoma in a subset of mouse prostate tissues [82,83]. Furthermore, the importance of estrogens in prostate carcinogenesis was also demonstrated by administrations of testosterone and estradiol to aromatase knockout mice that prevent conversion of testosterone into estradiol [84]. The combined treatment induced PIN whereas no change was observed in the mice with androgen alone, highlighting the implications of local production of estrogens within the prostate in the carcinogenesis [84]. In fact, increased expression of aromatase was frequently found in human PCa [85,86].

## 4. Estrogen Receptors (ERs) and Estrogenic Actions in Normal Prostate

It is widely believed that the actions of estrogens are mediated by ERs. Binding of estrogens leads to conformational changes of ER and then the complexes recruit transcriptional factors to form transcriptional machineries for gene expression. Next, the whole complexes bind to specific regulatory regions of DNA to stimulate transcription of estrogen-related genes such as progesterone receptor and pS2 [87,88]. Besides, ERs also regulate transcription through protein–protein interactions with other transcription factors such as AP-1 and Sp-1 to mold the transcriptional networks [89,90,91]. Ligand-binding studies demonstrated two estradiol-binding sites, including high-affinity (type I) and low-affinity (type II) sites, in the rat and human prostates. The type I-binding site usually refers to classical estrogen receptor (ERα) while the type II-binding site is still poorly characterized [92,93,94,95]. The combined treatment of testosterone and estradiol in Noble rats elevated the level of type II-binding site in the prostate along with an increase in wet weight of the glands [67]. Inhibition of this type II-binding site with a specific antagonist, 2,6-bis((3-methoxy-4-hydroxyphenyl)-methylene)-cyclohexanone, in intact adult mice reduced prostatic weight in a dose-dependent manner [96]. Fluorographic studies with [^3^H]-luteolin-labeled type II-binding site from rat uterine nuclear extracts identified a histone H3–H4 dimer likely responsible for the binding [97]. In addition, another estrogen receptor (ERβ) was cloned in rat prostate [98] and later found in human testis [99]. However, this ER subtype shows high affinity to estrogens similar to ERα. Taken together, the normal prostate contains at least two high-affinity estradiol-binding sites (ERα and ERβ) and one low-affinity (type II) site.

**Figure 1:**
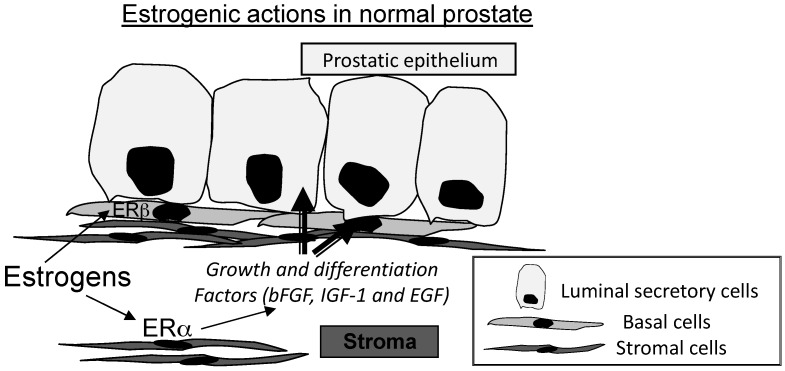
Estrogenic actions in normal prostate. Estrogens bind ERα in stromal cells to induce conformational changes of the receptor and the whole complexes stimulate the cells to express stromal cell growth and differentiation factors like basic fibroblast growth factor (bFGF), insulin like growth factor-1 (IGF-1) and epidermal growth factor (EGF) to regulate the growth and functions of prostatic epithelial cells in a paracrine action between stromal and epithelial cells. In addition, estrogens also exert direct effects on prostatic basal cells via interactions with ERβ to mold the estrogenic signals in the prostate.

Prior to the discovery of ERβ, immunohistochemical and in situ hybridization data demonstrated expression of ERα in the stroma of human prostate but not in epithelial cells [100,101,102]. During human fetal development, ERα is restricted to stromal cells [103]. Based on the cellular localization of ERα, estrogens were speculated to bind the stromal cell receptors and exert its indirect effects on epithelium via paracrine mediators like stromal cell-growth factors [104,105,106] (Figure 1). This notion was supported by the findings that the level of stromal basic fibroblast growth factor (bFGF) increased in the prostates of estrogen-treated rats [107]. In addition, estrogens enhanced synthesis of epidermal growth factor (EGF) and insulin like growth factor-1 (IGF-1) via an ER-mediated pathway to excite prostatic enlargement as demonstrated in organ culture of rat fetal prostate [108]. In line with these observations, these growth factors can stimulate growth of prostatic epithelium [109,110]. Administration of EGF or bFGF orthotopically in ventral prostates of adult rats increased the prostate size with enhanced growth of prostatic epithelium [109]. Moreover, systemic administration of IGF-1 can induce growth of the rat prostate [111]. It was also demonstrated that IGF-1 stimulated proliferation of prostatic epithelial cells derived from monkey prostate on extracellular matrix substrates [110]. However, there was no marked phenotype in the prostate of ERα knockout mice, suggesting that ERα is dispensable for the normal growth and functions of the prostate and the other compensating receptors also mediate estrogenic actions of the gland [112,113].

Discovery of ERβ in prostatic epithelial cells weakens the notion of sole paracrine estrogenic effects of stromal cells on prostatic epithelium. Estrogens also exert direct effects on epithelium of the prostate via ERβ. Previously, expression and cellular localization of the two ERs had been studied in normal human prostate but the results remained inconclusive. Bohkhoff and colleagues found that ERα expression was restricted to stromal and basal cells and the receptor was undetectable in secretory luminal epithelial cells. Also, none of stromal and epithelial compartments expressed ERβ [114]. In contrast, we [115] and others [116] demonstrated presence of ERβ only in basal epithelial cells, whereas ERα was predominantly expressed in stromal cells of normal human prostate (Figure 1).

Histologically, human prostate is comprised of peripheral, transitional and central zones and they have different hormone receptor profiles determined by ligand-binding assays [117,118,119]. High ratio of androgen receptor (AR) to progesterone receptor was found in transitional and central zones as compared to that in peripheral zone of the human prostate [117]. Uneven distribution of AR was also observed in normal human prostate, showing higher levels in the peripheral zone than those in the other zones [118]. Additionally, the transitional zone contains higher levels of EGF and bFGF as well as androgens than peripheral zones [120]. Different zones of the prostate showed differences in their tissue architectures, cellular morphology and hormone receptors profiles. The majority of prostate cancers are derived from the peripheral zone where PIN lesions are frequently found while benign hyperplasia frequently develops in the transitional zone. The central zone is rarely the site of PCa origin [121]. In the peripheral zone, basal and secretory cells in low- to moderate-grade dysplastic lesions expressed nuclear ERβ but the expression diminished in the dysplastic cells in the high-grade lesions [115]. No ERα-positive dysplastic cells were found in the peripheral zone of the prostate. In contrast, some dysplastic cells in the central zone showed ERα expression [115], possibly implying the biological differences and distinct pathways of hormone responsiveness between these two zones of the prostate [115].

## 5. Estrogen Receptor (ER) Expression and Estrogen Actions in PCa

ER expression in human PCa is also controversial. By immunohistochemistry and in situ hybridization, no ER expression was detected in cancerous epithelial cells, and ER mRNA/protein-expressing cells were limited to fibroblasts, myoblasts and smooth muscle cells of the tumor tissues [122,123,124]. On the contrary, Konishi et al. [100] detected a subset of ER-expressing PCa cells by immunohistochemistry. In addition, the results of RT-PCR demonstrated detectable levels of ER transcripts in two prostate cancer cell lines (PC-3 and LNCaP) that derived from PCa metastases [125,126]. Later, Bonkhoff et al. detected ERα but not ERβ transcripts in 11% of high-grade prostatic intraepithelial neoplasia, 43% of Gleason grade 4 PCa, and 61% of grade 5 cancer as well as 94% of recurrent adenocarcinoma after hormonal therapy [114]. However, Royuela and colleagues [116] observed both ERα and ERβ in epithelial compartment of the cancer. The stromal cells expressed ERα in the prostate but only a subset of PCa showed ERβ expression in their stromal compartments [116]. By RT-PCR of ERα, three PCa cell lines (LNCaP, ARPCa and C4-2) expressed ERα mRNA as well as its splicing variants [127]. For the clinical specimens, Latil and colleagues [128] demonstrated detectable levels of ERα and ERβ transcripts in both normal and cancer tissues and they found downregulation of ERβ mRNA expression in one half of either localized or hormone-refractory tumors. By immunohistochemistry and RT-PCR on the micro-dissected samples, significant reduction of ERβ expression was indicated in grade 4/5 carcinomas while the majority of grade 3 carcinomas expressed ERβ in the peripheral zone of the prostate [115]. In the extended cohort, ERβ expression was found in a few localized cancers and the expression was associated with poor relapse-free survivals [129]. In light of the findings of ERβ in sustaining E-cadherin expression and preventing an epithelial-mesenchymal transition (EMP) in PCa cells by sequestering Snail 1 in the cytoplasm [130], downregulation of this receptor expression in grade 4/5 carcinomas provokes acquisition of mesenchymal characteristics and aggressive behaviors of the cells that are associated with high-grade PCa.

Interestingly, the androgen-independent metastatic carcinoma cells in bone lesions and also nonosseous metastases regained ERβ expression, providing a critical foundation for developments of PCa therapy targeting this receptor for patients with relapses after androgen ablation therapy [115,131]. The mechanism underlying this regain remains unclear. As bone fibroblasts produced growth factors that, in turn, induced human PCa growth [132], it was speculated that the regain might be attributed to the unidentified stimulations of surrounding cells in the new tissue microenvironment of metastatic cells [115].

There are at least five splice variants of ERβ sharing the same first four functional domains but with different ligand-binding domains [133]. ERβ3 expression is restricted to testis whereas ERβ1, ERβ2, ERβ4, and ERβ5 are expressed in the prostate [133,134]. ERβ2 is the dominant isoform in PCa with no detectable affinity for estrogens and it is commonly localized in the cytoplasm, whereas the full-length ERβ1 is found in the nucleus [135]. It was demonstrated that ERβ2 functions as a transcriptional repressor of ERβ1 to negatively modulate ERβ1 activities [136]. Moreover, ERβ2 suppresses ERα signaling through proteasome-dependent degradation of ERα [137]. Besides cytoplasmic ERβ2, patients with biochemical relapse, postoperative metastases and shorter metastasis-free survivals frequently showed co-expression of nuclear ERβ2 and cytoplasmic ERβ5 in their tumors [135]. In line with this finding, ectopic expression of ERβ2 or ERβ5 in PCa cells enhanced their cell invasiveness, implying the metastasis-promoting roles of these two ERβ isoforms in PCa [135].

## 6. Mechanistic Insights of ERβ- and ERα-Mediated Signals

The first human ER was cloned in 1986 [138,139]. A decade later, the second ER was identified by screening a rat prostate cDNA library using the PCR products generated from degenerate primers recognizing the conserved regions within the DNA- and ligand-binding domains of nuclear receptors [98,140]. The second ER was named as ERβ and the original ER as ERα [98]. Then, the sequences of ERβ in different species including human and mouse were subsequently cloned [99,141]. Comparison of the amino acid sequences of these two receptors in rat, mouse and human showed that ERβ is highly homologous to ERα. They share 95%–97% homology in their DNA-binding domains and only 55%–60% in the ligand-binding domains. High variation was found in their N-terminal A/B domains with about 16% homology [98,99,141]. These differences likely result in differential cognate ligand profiles and transcriptional properties of these two receptors and elicit the uniqueness of each receptor in their biological functions.

Upon binding to ligands, ERs change their conformations and dimerize. The whole complex with other transcriptional factors interacts with specific DNA region as estrogen-responsive element (ERE) to regulate target gene expression. The ERE had been extensively studied in promoters of vitellogenin in Xenopus and chicken, and prolactin, progesterone receptor as well as pS2 in mammals. The consensus ERE consists of a 5 bp palindrome with a 3 bp spacer: GGTCAnnnTGACC [142]. By RT-PCR, tissue distribution of two receptors in rats had been investigated [143]. Kidney and adrenal gland predominantly or only express ERα transcripts while ERβ predominantly in lung, spinal cord and prostate. Both receptors are highly expressed in ovary and uterus [143]. In the ERα- and ERβ-coexpressing cells, the receptors predominantly form heterodimers upon binding to ligands as demonstrated by gel shift assays that two murine ERs dimerize with each other and bind to ERE [144]. The heterodimerization of human ERα and ERβ was also shown by mammalian two-hybrid assay and these physical interactions was verified by GST-pulldown and co-immunoprecipitation assays [145,146].

At estrogen responsive element (ERE), ERs employ two transactivation functional domains (AF-1 and AF-2 in N-terminal domain (NTD) and ligand-binding domain (LBD) respectively) to recruit p160 coactivator proteins such as steroid receptor coactivator-1 (SRC-1), glutamate receptor interacting protein 1 (GRIP1) and nuclear receptor coactivator 3 (NCO3) [147,148] that bind to an integrator molecule cyclic adenosine monophosphate response element-binding protein-binding protein (CBP)/p300. Crystallographic studies indicated that nuclear receptor boxes of p160 bind to the hydrophobic cleft on the surface of LBD of ERα [148,149]. Crystal structures of LBD of ERβ with ligands were similar to that of ERα and it also forms a hydrophobic cleft for binding of p160 coactivators [148,150]. The orientations of helix 12 of both receptors dictate the activities of ligands, either agonistic or antagonistic, at ERE though modulating the binding of p160 coactivator [148,149,150,151]. In contrast to AF-2 in LBD, the activation of AF-1 in NTD of two receptors is ligand-independent and is triggered by phosphorylation [148,152,153,154]. The domain binds to C-terminus of p160 coactivators such as GRIP1 and synergizes with AF-2 for transcriptional regulation [148,155].

In addition to ERE, ERs also regulate genes with promoters containing activation protein-1 (AP-1) sites [88,148,156,157]. The receptors, even without the DNA-binding domain (DBD), are able to transactivate ovalbumin gene promoter with AP-1 proteins like c-fos and c-jun proteins [156]. Similarly, IGF-1 expression is transcriptionally regulated by ER but there is no conventional ERE in the gene promoter [157]. ER facilitates binding of fos–jun protein complex onto the AP-1 site in this promoter for transactivation [157]. In collagenase gene promoter, tamoxifen- or estradiol-bound ER activated the promoter at the AP-1 site in both DBD-dependent and independent manners respectively [88,147].

Estrogenic activation at the AP-1 site requires integrity of both AF-1 and AF-2 [147,148]. The LBD of ERα strongly activates AP-1 promoter in the presence of estrogens but not tamoxifen [147]. Deletion or mutation of AF-2 or AF-1 abolished or severely reduced this activation [147]. However, the role of DBD is not quite crucial [147]. These indicated that the estrogenic activation at the AP-1 site is AF-1/2-dependent and DBD-independent as well as ligand-specific (i.e., estrogen but not tamoxifen) [147,156]. In this signaling, the AP-1 site recruits fos–jun complex that stimulates transcription by interacting with CBP/p300 and p160 coactivators. The estrogen-bound ERs trigger p160 in the pre-existing complex at the AP-1 site into a higher state of activity through both AF-1 and AF-2 of the receptors and then enhance CBP/p300 transcriptional activity [147].

On the contrary, tamoxifen-induced activation by ER at the AP-1 site was DBD-dependent and AF-independent [147,157]. Direct binding of ER onto the AP-1 site was not involved [157]. Deletion of DBD in ER abolished the promoter activation of IGF-1 [157]. ERβ bound with fulvestrant (ICI-182,780), raloxifene or tamoxifen but not estrogens can transactivate the promoter at the AP-1 site and the transactivation is completely independent of AF-2 [147,158]. ERα without AF-1 remained functional in this transactivation [147]. It indicated that AF-1 was dispensable in this DBD-dependent activation. As ER binds to a transcriptional corepressor N-CoR only in the presence of tamoxifen [159,160], Kushner and his colleagues proposed the mechanism for this DBD-dependent and AF-independent activation of ER upon binding with tamoxifen at the AP-1 site [147]. The tamoxifen-bound ER at the site, where it is away from the AP-1 site, binds to N-CoR and other corepressors and then the complex recruits histone deacetylases (HDACs), sequestering HDACs away from the AP-1 site. Eventually, the activity of histone acetylases in the fos–jun–p160–CBP/p300 complex is released, resulting in promoter transactivation [147,148].

In addition, tamoxifen-bound ER can stimulate human quinone reductase (QR) gene expression via electrophilic responsive elements (EpRE) [161,162]. EpRE motif in human QR gene consists of 12-o-tetradecanoylphorbol-13-acetate (TPA) responsive element (TRE), TRE-like element and AP-1 site. The ER-mediated regulation of QR expression is independent of AP-1 proteins because TPA, a potent AP-1 activity inducer [163], alone cannot transactivate QR transcriptional activity [161,162]. Gel shift assay with rat glutathione-s-transferase-Ya EpRE, which has no AP-1 site, showed that the major EpRE-interacting and activating proteins are not AP-1 proteins (i.e., the fos–jun complex) [164,165]. At EpRE, ERs interact with hPMC2, human homolog of the Xenopus gene (XPMC2), that directly binds onto the site to recruit Nrf2 and PARP-1 coactivators for upregulation of QR expression [166,167]. ERβ, rather than ERα, is the predominant interacting protein for hPMC2 [167]. Functionally, hPMC2 potentially acts as a negative cell cycle regulator through its regulation of QR transcriptional activity [166]. The enzyme activates the anticancer quinones which enhance cellular levels of reactive oxygen species (ROS) [168]. In turn, the increased ROS induced p21 expression to control cell cycle progression [168].

Interestingly, fulvestrant upregulated expression of interleukin-8 through NF-κB element of the promoter in a PCa cell line DU145 that predominantly expresses ERβ but no detectable ERα [169,170]. Upon fulvestrant treatment, ERβ interacted with p65 targeting the NF-κB site to stimulate the expression. It implicated the roles of ERβ in modulating NF-κB-mediated pathways [171].

In summary, ERs upon ligand binding regulate expression of target genes through homo- and/or hetero-dimerization and then directly binding onto ERE to transactivate the promoters or physically interacting with other transcriptional factors like AP-1, hPMC2 and NF-κB to recruit co-activators/co-repressors to mode the actions of estrogens and/or antiestrogens in a cell type-specific manner. Through these signaling actions, the endogenous and exogenous estrogens via ERs elicits their roles in controlling cell proliferation, apoptosis, migration and invasion capacities and communication networks with other cells. For instance, both AP-1 and hPMC2 are important in transcriptional regulation of p21 expression, in turn controlling the cell growth [156,168]. Also, regulation of hPMC2 expression by ERs in the cells modulates ROS levels in response to stress [166,167], and excess ROS can induce apoptosis. By interactions of ERβ with p65 targeting NF-κB site [170], estrogenic signaling cross-talks with NF-κB pathway to control apoptosis and also cellular and tumor immunity. Any inappropriate stimulations of this signaling could tip the balance of these estrogenic signaling networks and then the related cellular processes, resulting in transformation into cancer/diseased cells.

## 7. Biological Functions of ERα and ERβ Derived from Their Knockout Mice Models

Precise disruption or knockout of a particular gene in animals and examination of their phenotypes can provide great insights into the roles of the gene in development and normal physiology. ERα [171,172] and ERβ [172,173] single knockout (ERαKO and ERβKO) and double knockout (ERαβKO) [172,174] mice were generated and all of these KO mice showed no lethality. Their phenotypes were distinct [172,174,175]. Males and females of ERαKO and ERαβKO mice are infertile while only ERβKO females are either infertile or subfertile with reduced litter size and the males are fertile [171,172,173]. In the female reproductive tract, ERαKO mice develop hypoplastic uterus and vagina and there is no cyclic change [171]. However, the genital tract of ERβKO is normal [173]. The lengths of oviduct, uterus horns and vagina of ERαβKO are normal while diameter and thickness are smaller than those in wild-type females [172,174]. Ovaries of ERαKO adults are anovulatory. They developed multiple hemorrhagic cysts and no corpora lutea. The ovaries underwent normal prenatal and neonatal developments [171,172]. Similar features of ovaries were found in ERαβKO females. All ERαβKO ovaries contained fully differentiated Sertoli cells and exhibited follicle transdifferentiation to structures resembling seminiferous tubules of the testis [172,174]. They also showed increases of atretic follicles [173]. However, an independent study of ERαβKO mice found no such change and the ovaries were macroscopically normal [172]. In most of them, corpora lutea were scarce or absent [172]. These findings indicated that the roles of both ERα and ERβ in proliferation of granulosa cells are crucial and the presence of these two receptors are required to complete the folliculogenesis in ovary [172]. In the early stages of folliculogenesis, two ERs apparently are dispensable and can partially compensate each other in the inactivation of either of these two receptors. They showed some degrees of functional redundancy [172,174].

In ERαKO and ERαβKO males, the testes lack germ cells in the seminiferous tubules and show a marked dilation of straight tubules and rete testis [141,174]. The male reproductive tracts of ERβKO mice are normal, but the older animals develop hyperplasia in the prostate and the urinary bladder [173]. However, no such abnormalities were reported in a subsequent study and it even showed no change in cell proliferation in the prostates of 8- and 20-month-old ERαβKO mice with labeling indices by Ki67 and BrdU immunostainings [172]. Instead, the authors reported abundance-to-massive lymphoid aggregates associated with reactive epithelium in the prostates of 12-month-old ERβKO mice and suggested the immunomodulatory roles of prostatic ERβ [176].

## 8. Estrogen Therapy for Prostate Cancer

Hormonal therapy is the mainstay of treatment in patients with metastatic PCa. Medical castration is commonly accomplished with luteinizing hormone-releasing hormone (LHRH) analogs. Diethylstibestrol (DES), a synthetic estrogen, is a good alternative for cost-effective therapy [177,178,179,180,181]. Theoretically, DES suppresses LHRH-induced stimulation of the pituitary gland and thus indirectly reduces production of testosterone in testis, resulting in decrease of serum testosterone to the anorchid level [178,179,182]. Subsequently, the androgen-dependent PCa regresses. Unfortunately, the disease eventually progresses into androgen-independent cancer that is irresponsive to this androgen ablation therapy. Emerging evidence indicated that DES did not suppress cancer growth solely through this pituitary–gonadal axis. Treatment of DES in the orchidectomized patients who already had very low levels of serum testosterone is still effective in suppression of PCa growth, suggesting that the inhibitory effects of DES are not only attributed to the reduced testosterone production. Several studies showed direct actions of DES on PCa cells [181,183,184,185]. Robertson et al. [185] demonstrated direct cytotoxic effects of DES on PCa cells via induction of apoptosis while Hartley-Asp et al. [184] indicated DES-induced metaphase arrest and inhibition of microtubule assembly in the cells. In addition, in vitro studies highlighted the involvements of ER in the estrogen-induced inhibition of PCa cell growth [125,126]. In contrast, estrogens, especially 17β-estradiol (E_2_), stimulated proliferation of androgen-responsive PCa LNCaP cells, possibly related to the mutated androgen receptor with threonine to alanine at codon 868 which has high affinity to estrogens [186,187]. However, presence of ER by immunohistochemistry and RT-PCR analysis and blockade of E_2_-induced growth by antiestrogen in LNCaP cells suggested the biological responses of LNCaP cells to E_2_ is likely mediated via ER [126]. For the androgen-irresponsive PCa cells (PC-3 and DU145), E_2_ inhibited the cell growth through an ER-associated pathway [125,169].

## 9. Potential of Antiestrogens for PCa Therapy

**Figure 2:**
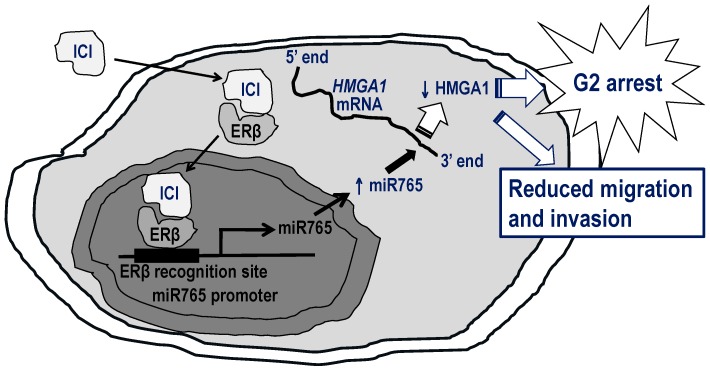
The proposed mechanism underlying the inhibitory effects of fulvestrant (ICI) on prostate cancer cells. Fulvestrant (ICI) interacts with ERβ and the complexes translocate into the nucleus to transactivate the promoter of hsa-miR765 at the ERβ recognition site. Upon stimulation, the level of hsa-miR765 increases and the miRNAs recognize the 3′-UTR of its target HMGA1 transcripts to predominantly suppress the translation and, at least in part, reduce the transcript stability, resulting in reduction of HMGA1 protein expression in the cells. Eventually, the reduction induces G2 cell cycle arrest and interferes with the metastatic potential of the cells.

In the early 1980s, Noble [188] demonstrated the estrogen-dependence of PCa in Noble rat model. Removal of estrogen treatment and also blockade of estrogenic actions by a non-steroidal antiestrogen tamoxifen with continued estrogen treatment inhibited the tumor growth. These findings prompted the potential uses of antiestrogens as therapeutic agents for PCa [188]. Subsequently, various clinical trials were conducted to evaluate the efficacy of tamoxifen on patients with advanced PCa [189,190]. They demonstrated tamoxifen to be a palliative therapeutic agent for the disease, partially due to its estrogenic properties [191,192,193]. In the Eastern Cooperative Oncology Group trial, no patient responded to the treatment [194]. Moreover, the estrogenicity of tamoxifen posed increased risks of cardiovascular disease similar to the side effects of DES treatment. Therefore, evaluations of the potential uses of antiestrogens as therapeutic agents in a single-arm or adjuvant treatment for PCa patients were discontinued. It was not until later that two steroid antiestrogens, ICI-164,384 and ICI-182,780 (fulvestrant), were developed with no estrogenic activity, different from tamoxifen [195,196]. The therapeutic potential of these antiestrogens for PCa were revisited and preclinical studies demonstrated anti-proliferative effects of fulvestrant on androgen-responsive and -irresponsive PCa cells (LNCaP, DU145 and PC-3) [169,197]. Fulvestrant inhibited androgen-responsive LNCaP cell growth through downregulation of AR expression [197], whereas ERβ mediated the inhibition in androgen-irresponsive and AR non-expressing DU145 cells through modulations of NF-κB signaling [170] and upregulation of hsa-miR-765 expression to suppress oncogenic HMGA1 protein expression [198] (Figure 2). Regarding the clinical relevance, the ERβ-hsa-miR-765-HMGA1 signaling axis was also observed in the patients treated with fulvestrant [198]. Furthermore, the drug impaired cell migration and invasion of PCa cells possibly via reduction of filopodia/stress fibers formations, providing the mechanistic insights of how fulvestrant inhibited PCa cell growth and its metastatic behaviors [198] (Figure 2).

In the early clinical trial of fulvestrant in castration-resistant PCa (CRPC) with a single dose of 500 mg on day 0 and then 250 mg on day 14, day 28, and monthly thereafter for six months in 20 patients, the treatment was well-tolerated but no clinical or PSA response was obtained [199]. However, the subsequent trial with 500 mg fulvestrant every 14 days for the first month and 250 mg monthly thereafter in seven highly pretreated CRPC patients demonstrated initial reduction of PSA levels in six of these patients even though the levels increased after the dose was reduced to 250 mg [200]. This observation clearly implied dose-dependent responses of fulvestrant in PCa [200] and thus warranted further studies of dose optimization for fulvestrant therapy in PCa patients. Importantly, comparing with chemotherapy that is not always the option for elderly and fragile patients with co-morbidities, fulvestrant treatment with low toxicity profiles could be specifically beneficial to these patients. For high-dose treatment, the possible increased toxicity may hamper the continuation of the treatment in the patients. However, the improved therapeutic efficacy and no new safety concerns identified for high-dose (500 mg) fulvestrant treatment in recurrent and metastatic breast cancer patients compared with the low-dose (250 mg) treatment were reported [201]. These promising results should lead toward future trials on high-dose fulvestrant in PCa patients.

## 10. Significance of a G-Protein-Coupled Receptor GPR30 and Its Therapeutic Potential in PCa

In addition to receptor-mediated transcriptional regulations of target genes in estrogenic actions, estrogens are able to trigger rapid signaling cascades initiated at the plasma membrane [202,203]. They mediated through activations of MAP kinases, PI3K kinase, protein kinase C and protein phosphatases and/or through releases of intracellular cAMP, cGMP and calcium [202,203]. By ligand-binding studies using radioactive labeled estrogens or membrane-impermeable fluorescent dye-labeled estrogen conjugated with bovine serum albumin (E_2_-BSA conjugates), estrogen-binding sites were demonstrated to be at the plasma membrane [204]. In addition, these rapid estrogenic signaling responses were stimulated by membrane-impermeable E_2_-BSA conjugates, independent of cytosolic ERs [205,206,207,208]. Previous studies also suggested that there were approximately 5%–10% of cellular ERα and/or ERβ and their splice variants at the plasma membrane, instead of in cytoplasm, responsible for these non-genomic estrogenic responses [209,210,211,212,213].

Besides ERα and ERβ, an orphan G-protein-coupled receptor (GPR30) showed high affinity but low-capacity binding to estrogens/phytoestrogens/antiestrogens/selective estrogen receptor modulators. It was detected at the plasma membrane and endoplasmic reticulum, also referred to as the G-protein-coupled estrogen receptor (GPER) [214,215,216,217]. It belongs to the family of 7-transmembrane G-protein-coupled receptors [218]. It was speculated that GPR30 together with ERα and/or ERβ elicited non-genomic estrogenic responses initiated at the plasma membrane in the estrogen-targeted cells [219]. By estrogenic stimulation, the receptor induces rapid but transient Erk1/2 activation to promote cell proliferation in ERα/ERβ-negative breast cancer cells as well as in endometrial, ovarian, and thyroid cancer cells [220,221,222]. On the contrary, GPR30 activation suppressed in vitro growth of normal and malignant bladder urothelial cells [223]. We also demonstrated the inhibitory functions of this receptor upon activation in PCa in both in vitro and in vivo models [224]. These implied the dual roles of GPR30 in controlling cell growth in a cell type-specific manner.

A number of estrogenic responses that are mediated through ERα and ERβ had been reflected by the phenotypes of the single and double knockout mice of these two receptors (ERαKO, ERβKO and ERαβKO) [171,172,173,174,175]. However, some estrogen-dependent phenotypes, independent of ERα and ERβ, were observed in ERαβKO mice, highlighting the presence of additional receptor-like GPR30 for estrogen-dependent physiology [225,226]. In ERαβKO mice with no ERs, pancreatic β-cells retained estrogenic protection from apoptosis [225]. Knocking out of GPR30 in these mice impaired the protective effects and they were susceptible to apoptotic stress in pancreatic islet, implicating the significance of GPR30 in this estrogenic protection [225].

Regarding GPR30-mediated signaling, its involvements mainly in cell-growth stimulation had been studied in various estrogen-related cancers since Filardo et al. [214] first described the estrogen-mediated and GPR30-dependent activation of MAP kinase Erk1/2 via transactivation of epidermal growth factor receptor in breast cancer cells. Estrogen 17β-estradiol and antiestrogen fulvestrant (ICI-182,780) induced a rapid but transient activation of Erk1/2 in ERα/ERβ-negative breast cancer cells SKBR3 that express GPR30 but not in GPR30 non-expressing MB-MDA231 cells [214]. It is mediated through Gβγ-mediated signaling as Gβγ sequestrant peptide inhibited the activation [214]. For mechanisms underlying the rapid restoration of Erk1/2 activity to the baseline level, Filardo et al. [227] demonstrated that estrogen via GPR30 simultaneously activated adenylyl cyclase (AC) to increase intracellular cAMP that eventually attenuates Erk1/2 activity, then resulting in cell-growth stimulation in the cells.

To adequately delineate the GPR30-mediated signaling, identification of G-1 (1-[4-(6-romobenzo[1,3]dioxol-5yl)-3a,4,5,9b-tetrahydro-3*H*-cyclopenta[c]quinolin-8-yl]-ethanone) by virtual and biomolecular screening approaches as a GPR30-selective agonist [228] is critical, especially for studying the functional roles of GPR30 in the cells that co-expressed ERα and/or ERβ. Importantly, G-1 displayed no significant binding to either ERα or ERβ [228]. Thus, G-1 specifically activates the GPR30-mediated signaling without interferences of ERα- and/or ERβ-associated complications. In BG-1 ovarian cancer cells and SKBR3 breast cancer cells, G-1 stimulated cell proliferation through transient activation of Erk1/2 and induction of c-fos and cyclin D1 expression [222].

GPR30-mediated signaling for cell-growth inhibition is relatively less understood. In human bladder urothelial cells, G-1 inhibited cell growth but failed to induce c-fos and cyclin D1 expression [223]. However, estrogen stimulated urothelial cell growth with induction of these genes expression [223]. Overexpression of GPR30 abolished the estrogen-induced c-fos and cyclin D1 expression, whereas siRNA knockdown of this receptor increased gene expression, suggesting that GPR30 reduced proliferative effects of estrogens on urothelial cells by hampering the estrogen-induced genes expression [223].

For PCa, we reported dose-dependent cell-growth inhibition by G-1 through GPR30-dependent G2 cell cycle arrest [224]. The sensitivity to G-1 depends on the level of GPR30 in PCa cells as the IC50 for high GPR30-expressing PC-3 cells is lower than that for low GPR30-expressing DU145 cells [224]. Expression of GPR30 obtained from gene expression microarray databases of Vanaja et al.’s [229] and Varambally et al.’s [230] studies and also our cohort of samples [224] showed variations in the expression among clinical samples with slight reduction in PCa as compared to normal tissues, suggesting that the sensitivity to G-1 among patients may be different and expression status of GPR30 could be used as a biomarker for prediction of a patient’s outcomes for GPR30-targeted therapy in future clinical trials.

**Figure 3:**
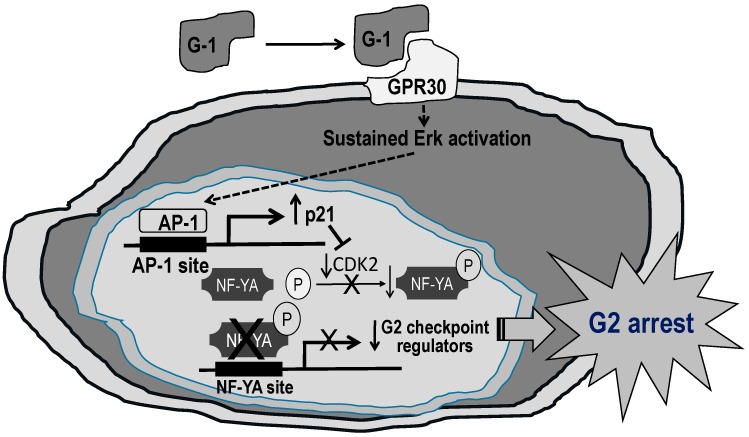
The proposed mechanism underlying the inhibitory effects of GPR30 activation by G-1 on prostate cancer cell growth. Upon GPR30 activation by G-1, Erk1/2 are activated with nuclear translocation of phosphorylated Erk1/2. The activation is sustained for up to four days. The sustained Erk1/2 activation stimulates AP-1 proteins (i.e., increased levels of phosphorylated c-fox and c-jun) to induce p21 expression. As p21 is a negative regulator of CDK1 which activates NF-YA, the increased p21 reduces the levels of CDK1 and active NF-YA, resulting in downregulation of G2 checkpoint regulators expression including CCNA, CCNB, cdc2 and cdc25C to induce G2 cell cycle arrest and eventually inhibit the cell growth.

Mechanistically, activation of GPR30 by G-1 inhibited PCa cell growth through sustained activation of Erk1/2, c-jun/c-fos-dependent upregulation of p21 expression, and downregulation of G2-checkpoint regulators (cyclin A2, cyclin B1, cdc25c, and cdc2) expression by reduced phosphorylation of their common transcriptional factor NF-YA, resulting in arrest of PCa cell growth at G2 phase [224] (Figure 3). In addition, G-1 inhibited growths of both androgen-dependent and androgen-independent PCa cells in vitro and PC-3 xenografts in vivo [224]. Importantly, G-1 elicited no growth or any histological change in the prostate of intact mice and did not affect cell growth of quiescent BPH-1, an immortalized benign prostatic epithelial cell line, indicating the safety of this therapy [224]. The therapeutic actions of G-1 and their relationships with androgen in vivo was investigated using the LNCaP xenograft to model PCa growth during the androgen-sensitive (AS) versus the castration-resistant (CR) phase recapitulated the natural history of PCa progression 231]. GPR30 expression was reduced by androgen via AR and castration upregulated the expression [231]. In human specimens, metastatic CRPC expressed a high level of GPR30 [231]. Regarding the therapeutic actions of G-1, this GPR30 agonist specifically inhibited CR but not AS tumors with marked neutrophil infiltration in the affected tumors and low risk of toxicity [231]. These preclinical data lay a crucial foundation for further development of GPR30-targeted therapy for the patients.

It was well-established that duration of Erk1/2 activation dictates the cell fate with transient Erk1/2 activation leading to cell survival and proliferation whereas sustained activation transmitting antiproliferative signals [232,233]. In concordance, we reported that G-1 induced sustained nuclear accumulation of phosphorylated Erk1/2 in PC-3 cells but not in MCF-7 breast cancer cells that only showed transient accumulation [224]. Transient activation in breast cancer cells is attributed to the negative feedback via Gα-protein-mediated activation of AC [227]. However, the underlying mechanism about how to sustain Erk1/2 activation eventually to inhibit the growth of PCa cells remains unclear.

In addition to controlling cancer cell growth, GPR30 activation by antiestrogen tamoxifen upregulated expression of Connective Tissue Growth Factor (CTGF) via transactivation of its promoter by AP-1 to promote cell migration in SKBR3 breast cancer cells [234], shedding light onto the GPR30-mediated stimulatory effects on breast cancer cell metastatic potentials. In line with this observation, high expression of GPR30 correlates with clinicopathological biomarkers for poor outcomes and also with increased tumor size and metastasis in clinical breast cancers [235]. However, activation of GPR30 by its agonists including G-1, estrogen, fulvestrant and tamoxifen attenuates EGF-induced rapid mobilization of urokinase plasminogen activator receptor to inhibit migration of ovarian cancer cells [236]. Similar to cell-growth regulations, these opposite data also suggest the dual roles of GPR30 in controlling cell migration, depending on cell types and being mediated via different mechanisms. Whether and how GPR30 is involved in PCa cell migration and even invasion are yet to be determined. Future studies delineating the functional significance of GPR30 and its underlying mechanisms in governing these metastatic behaviors of PCa are warranted.

Recently, doubts about the specificity of G-1 have emerged. Instead of binding to GPR30, G-1 was demonstrated to directly interact with ERα36, a 36 kDa membrane-localized splice variant of ERα, to activate Erk1/2 signaling in SKBR3 cells [237]. In this activation, GPR30 is involved in controlling ERα36 expression [237]. It prompts a concern about the exact molecular target of G-1 in cell signaling. Future pharmacological developments of more specific GPR30 agonists and antagonists are needed to reveal the exact functional and mechanistic roles of this receptor in PCa and also other estrogen-related cancers.

## 11. Conclusions

Nearly three-quarters of a century has passed since the first demonstration of androgen dependence of PCa by Huggins and Hodges, leading to the eventual establishment of androgen ablation therapy for patients. This therapeutic approach remained as the mainstay PCa treatment for several decades. Throughout these years, a tremendous amount of research efforts disclosed the importance of estrogenic and antiestrogenic signaling via their receptors like ERα, ERβ and GPR30 in controlling PCa growth and its metastatic phenotypes. Mechanistically, the molecular and functional consequences of these signals in PCa have been investigated and gradually revealed. Yet, the exact molecular networks of these estrogenic and antiestrogenic signaling in PCa have not been fully deciphered. The roles of ERβ in preventing EMT in PCa due to downregulation of this receptor expression frequently found in high-grade cancers highlighted the importance of this receptor in controlling PCa aggressiveness. Thus, the mechanism underlying this downregulation and approaches for hindering the alteration could be the direction for future study. Regarding GPR30, the current preclinical data demonstrated the therapeutic potential of the G-1-induced GPR30 activation for PCa. The therapy efficacy and toxicity should be further evaluated in clinical trials for metastatic CRPC patients. Furthermore, there are emerging concerns about the specificity of G-1, the identification of more specific derivatives could therefore be beneficial for developments of this GPR30-targeted therapy.
